# Clinical use and applications of a citrate-based antiseptic lavage for the prevention and treatment of PJI

**DOI:** 10.3389/fmed.2024.1397192

**Published:** 2024-07-02

**Authors:** Daniel Alejandro Valdés, Jon E. Minter

**Affiliations:** ^1^Philadelphia College of Osteopathic Medicine (Georgia), Suwanee, GA, United States; ^2^Northside Hospital, Atlanta, GA, United States

**Keywords:** prosthetic join infection (PJI), arthroplasty, biofilm, orthopedic surgery, arthroplasty outcomes, arthroplasty complications

## Abstract

Total joint arthroplasties (TJA) are some of the most commonly performed surgeries in the United States with the number of TJA expected to rise significantly over the next decade as the population ages and arthritic burden worsens. However, the rise in TJA volume correlates with a heightened risk of complications, notably prosthetic joint infections (PJI), despite their low occurrence rate of less than 2%. PJI imposes a significant burden on surgery success, patient well-being, and healthcare costs, with an estimated annual expense of 1.85 billion dollars for hip and knee PJI by 2030. This manuscript delves into the pathophysiology of PJI, exploring our current understanding of the role of bacterial biofilm formation on implanted foreign hardware, providing protection against the host immune system and antibiotics. The article reviews current agents and their efficacy in treating PJI, as well as their cytotoxicity toward native cells involved in wound healing, prompting the exploration of a novel citrate-based solution. The paper highlights the superior properties and efficacy of a novel citrate-based irrigation solution on the treatment and prevention of PJI via increased antimicrobial properties, greater biofilm disruption, increased exposure time, and reduced cytotoxicity compared to conventional solutions, positioning it as a promising alternative. It also provides a perspective on its clinical use in the operating theater, with a step-by-step approach in TJA, whether primary or revisionary.

## 1 Introduction

Total joint arthroplasties (TJA) make up some of the most commonly performed orthopedic surgical procedures in the United States. The annual incidence of total knee arthroplasties (TKA) and total hip arthroplasties (THA) is over one million, with that number set to drastically increase by the year 2030 (1), likely secondary to a steady rise of arthritic burden in the aging population (2). The increase in the volume of TJAs is accompanied by an increased incidence of postoperative complications. Prosthetic joint infections (PJI) are one such potential complication, though rare in nature. Although PJI occurs at a rate of less than 2% (3), it represents a devastating burden to the success of the surgery, the physical and emotional well-being of the patient, and the healthcare system as a whole. Patients with PJI often require prolonged hospital stays and multiple revision surgeries, increased need for ambulatory aids, and reduced joint function scores, as well as poorer global quality of life (4). At the current trajectory, the annual hospital costs of PJI for the hip and knee are projected to be 1.85 billion dollars by the year 2030, placing a large burden both on the patient and the healthcare system as a whole (5).

## 2 Biofilm

Underlying the pathophysiology of PJI is the bacterial formation of biofilm. Understanding biofilm requires an understanding of why bacteria form this polymeric, variably dense structure in the first place. Ultimately, the “why” can be summarized by three primary incentives: (1) biofilm protects bacteria from harmful conditions in the host, (2) sequestration to a nutrient-rich environment, and (3) utilization of cooperative benefits (6).

Each point can be discussed individually to briefly explore these in more detail.

Biofilm protects bacteria from harmful conditions in the host. Unlike free-floating planktonic bacteria, sessile growth, which results in biofilm formation, provides microbes with a 1,000-fold increase in resistance against nutrient deprivation, pH changes, oxygen radicals, as well as against externally applied disinfectants and antibiotics that may be employed by the host to eradicate them (7). Underlying the seemingly impenetrable matrix that is biofilm lies a vital molecule that is produced by many different types of biofilm-forming bacteria: exopolysaccharide (EPS). For example, in gram-negative organism *Escherichia coli*, the gene csgA encodes for colonic acid, which is involved in aggregation. In Pseudomas aeruginosa, the algC encodes for alginate synthesis (8–10). Gram-positive organisms similarly code for and produce such aggregation molecules, as seen with GbpA coding for glucosyltransferase in *Streptococcus mutans* and icaADBC coding for B-1-6-linked poly-N-acetylglucosamine polymer (PNAG) seen in *Staphylococcus aureus* and *Staphylococcus epidermidis* (11, 12). The importance of EPS is notably seen in the difference in the biofilm morphologies of weak PNAG-producing strains versus strong PNAG-producing strains of *S. aureus*, where the former creates a naive, more vulnerable biofilm and the latter a mature, compact biofilm that is more resistant to shear forces (4).

Biofilm provides sequestration in a nutrient-rich environment. The human body provides an excellent environment full of water, oxygen availability, temperature, and nutrients for bacterial survival. In fact, previous studies have shown that EPS expression and biofilm production are markedly enhanced in several types of bacteria, including pseudomonads, *V. cholerae*, *E. coli*, staphylococci, and streptococci, when glucose or readily available sources of carbon are abundant in its local environment. However, when the nutrients deplete, the bacteria become planktonic in search of another nutrient-rich environment (4, 13). Thus, the human condition can be viewed as a never-ending battle for balance between our immune system, commensal organisms, and invading pathogens constantly developing methods of evading said immune system.

Biofilm allows for the utilization of cooperative benefits, often consisting of a variety of bacterial species and even fungi, each performing different functions and promoting the strength and survival of the biofilm. This phenomenon of inter-species commensalism is observed in the oral cavity, where early on, aerobes and facultative anaerobes can survive in an oxygen-rich environment. Still, as biofilm forms and oxygen diffusion across the biofilm becomes limited, obligate anaerobes have been seen to colonize among the oral microbiome (11). Furthermore, even intra-species commensalism can be observed in biofilms through phenotypic variations within the same bacterial species. For example, in the context of antibiotic recalcitrance, persister cells are a particular subset of a bacterial species that comprise the biofilm, arising either through environmental triggers (Type I) or spontaneously (Type II), and they serve the sole purpose of providing tolerance against antibiotics via several mechanisms including the reduction of reactive oxygen species (ROS). Though these persister cells may eventually die after prolonged exposure, they serve the larger purpose of ensuring the survival of the biofilm (14). *B. cereus* produces highly resistant and adherent spores which greatly increases biofilm resistance to antimicrobial agents and disinfectants (15). Finally, from a reproduction standpoint, biofilms provide an ideal environment for horizontal gene transfer (16, 17).

## 3 Periprosthetic joint infections (PJI)

Foreign bodies provide a site of inoculation and subsequent seeding for bacteria. Several factors increase the adherence of these organisms to the surface of implants, some of which are native to the bacteria (e.g., adhesins). Other factors are intrinsic to the foreign material (e.g., surface tension, hydrophobicity, and electrostatic forces) and can be seen, for example, in the significantly greater rates of infection with the use of stainless steel implants versus titanium (18). Once seeded, the bacteria may accumulate and synergistically work together to mature a protective biofilm to create a favorable niche for their survival and reproduction. The creation of biofilm may also explain why PJIs can have a delayed or late onset and may also explain why even after treatment and revision surgeries, PJIs recur at a rate of 9–12% (19, 20).

## 4 Prevention of PJI

Extensive research endeavors persist in the development of strategies to prevent biofilm formation and reduce the risk of PJI. Historically, such efforts have included screening (14), intravenous antibiotics (21), wound closure approach/devices (22), and irrigation (23). Numerous studies have been published comparing the efficacy of existing irrigation solutions against biofilm (24–26). While we know that the use of irrigation is a critical step in the treatment of PJI (27), there is a lack of evidence and consensus upon which agent is superior, which is defined not only by antimicrobial activity and biofilm penetration but also minimization of cytotoxicity to human osteoblasts, myoblasts, chondrocytes, and fibroblasts, as these cells play a pivotal role in wound healing and the overall success of surgery (28). Some of the most commonly used agents have been shown to be cytotoxic toward these native cells, including povidone-iodine (PI) (29, 30), chlorhexidine gluconate (CHG) (24), and hypochlorous acid (31),but they all come with unique downsides.

Betadine releases PI, a powerful oxidizer to cell membranes, which has been shown to decrease the transcription of the *ica* gene in the staphylococcus species, thereby reducing the production of polysaccharide intercellular adhesin (PIA), one of the primary EPSs involved in biofilm formation (32). However, studies have shown PI to be cytotoxic to human fibroblasts, myoblasts, osteoblasts, and chondrocytes, necessitating its dilution for the minimization of harm. The optimal concentration of PI has not been found, but its commercially available concentration of 100 g/L (10%) has been shown to be both bactericidal and cytotoxic (33, 34).

CHG exists in the cation form at physiological pH, allowing it to bind to negatively charged bacterial membranes. Although broad in its antimicrobial spectrum of activity, multiple *in vitro* studies have shown that at concentrations as low as 0.02%, CHG may drastically affect local native connective tissue and stromal cells (24, 34).

Hypochlorous acid is a natural part of the immune response, which native white blood cells release to kill pathogens during oxidative bursts. It is found in commercially available forms for use for superficial wound management, including diabetic ulcers and burns (2), and has even been shown to combat *S. aureus* biofilm production with low cytotoxic effects on native cells (35). However, hypochlorous acid has been shown to have substantial erosive properties on cobalt-chromium and titanium metals (36), two of the most commonly used metals in arthroplastic implants (36).

As previously mentioned, biofilm production seems to be at the core of the pathogenesis of PJI; hence, a variety of irrigation solutions, some of which have been discussed here, have been tried and utilized by surgeons both in the setting of decreasing the risk of developing biofilm formation, as well as penetrating and preventing maturation of existing biofilm in debridement, antibiotics, and implant retention (DAIR) procedures or single/double-stage revision surgeries for the treatment of PJI.

## 5 A novel agent–citrate-based solutions

In general, wound dressings that are approved by the Food and Drug Administrtation (FDA) are biomaterials with naturally derived ingredients such as collagen and alginate, as well as synthetic polymers such as polylactic acid (PLA) and poly lactic-co-glycolic acid (PGLA). Not only do many of these commonly used biomaterials lack intrinsic antibacterial properties, but they may also harm native cells in the human body. For this reason, the search for more organic antiseptics that both effectively treat a broad-spectrum of bacterial infections and carry low burden on the host’s tissue has augmented within the last decade.

Although the application of citrate-based solution is relatively new in the field of orthopedics, it has been thoroughly studied in endodontics, and there may be plausible similarities in pathophysiology that make its application in the treatment of PJI not only possible but promising. Citrate has been historically used in the eradication of the smear layer produced during mechanical instrumentation in endodontic procedures (37). The smear layer, composed of dentine, remnants of pulp tissue, odontoblastic processes, and bacteria, is in many ways similar to biofilm (37, 38). Similarly, in the field of Orthopedics, it has been increasingly evident that a key aspect of both treatment and prevention of PJI revolves around penetration and eradication of biofilm formation (6). Throughout the rest of this paper, we will focus on why the use of citrate-based antiseptics is a superior alternative to other classically used irrigants and washes in the treatment and prevention of PJI, with a focus on the following points: (a) increased antibacterial properties, (b) greater biofilm disruption, (c) increased exposure time, (d) decreased cytotoxicity on native cells and host tissue.

## 6 Increased antimicrobial potency

Citrate-based solutions have been consistently used for its antimicrobial properties in endodontics (9). The mechanism of citrate’s antimicrobial activity may be multifaceted. While the antimicrobial activity of citrate involves the chelation of metal ions from the EPS matrix, it is also associated with a concurrent shift in the permeability of bacterial cell walls (1). These events may collectively contribute to cell death, especially in Gram-negative bacteria. For this reason, citrate is routinely used in the treatment of gram-negative infections, such as bismuth potassium citrate in the eradication of *Helicobacter pylori*. As an organic acid, citrate affects pH levels, potentially both in the local environment and intracellularly, interrupting enzymatic activities and DNA synthesis, leading to microbial death (39, 40).

Mani-Lopez et al. proposed that the nature of citrate an organic acid allows it to flow through cell membranes and lower intracellular pH, leading to the degradation of enzymatic processes, proteins, DNA, and the extracellular membrane (40). Kong et al. proposed that organic acids can lower the pH, suppressing nicotinamide adenine dinucleotide (NADH) oxidation (41). Yet another potential mechanism involves the alteration of the pH in the local environment, chelating metal ions in the bacterial cell wall, affecting its permeability and ultimately leading to cell death (39).

## 7 Greater biofilm disruption

In addition to antimicrobial properties, citrate-based solutions have shown an enhanced ability to penetrate, disrupt, and prevent biofilm formation. *In vitro*, Bashyal et al. demonstrated that a novel citrate-based solution, Xperience_TM_ ([XP] Next Science LLC, Jacksonville, FL), containing 32.5 g/L citric acid, 31.3 g/L sodium citrate, and 1.00 g/L lauryl sulfate in water, reduced biofilm burden of multiple bacterial strains including Staphylococcus, Pseudomonas, Cutibacterium, and E.coli by 4-log to 6-log, as compared to less than 1-log reductions observed using betadine, Irrisept (0.05% CHG), or Vashe (0.03% HOCL) (42).

An important consideration here is the addition of sodium lauryl sulfate (SLS) to the antiseptic compound. SLS, the sodium salt of lauryl alcohol, has been the most widely used of the denifrices for several decades. As a surfactant composed of a hydrophilic head and hydrophobic tail, it reduces surface tension and induces micellization of hydrophobic components and denaturation of proteins (43, 44). There are multiple mechanisms by which surfactants are proposed to inhibit biofilm formation, including interactions with cellular components, disruption of bacterial aggregation, as well as interference with inter-bacterial quorum sensing (45, 46).

While many surfactants are non-ionic in nature, SLS is anionic and has not only shown to be an effective drug vehicle, but has also shown improved antimicrobial properties compared to other surfactants, likely due to its shorter-chain fatty acid (47, 48). In fact, Sharma et al. explored the intrinsic capabilities of various surfactants in solubilizing lipid membranes in the context of killing gram-negative bacteria and found faster translocation times across peptidoglycans, greater hydrophobic mismatch and membrane thinning, and greater inner membrane poration in the presence of shorter-chain surfactants when compared to those with longer chains (47). However, when used alone, bacterial biofilm formation has been shown to develop recalcitrance toward detergent-stimulated detachment (Landa et al.). Hence, when combining an organic acid with strong antimicrobial properties such as citrate with a surfactant such as SLS, SLS is able to form micelles through encapsulation of bioactive materials and thereby adjust their hydrophilicity, allowing for easier and more effective penetrance of the antimicrobial (49). Previous works in the field of ophthalmology have further explored this synergistic effect, such as a study conducted by Doroshenko et al. assessed a highly-branched poly(N-isopropyl acrylamide) with vancomycin end groups significantly inhibited biofilm formation (*p* = 0.0008) on plastic and caused a 1-log reduction in infected rabbit corneas compared to controls (*p* = 0.002). Similarly, Karetsi et al. showed that a composite material composed of citrate dispersed in SLS reduced burden of *Staph aureus* up to 21-fold higher than citrate alone and up to 5-fold higher than SLS alone (49).

## 8 Increased exposure time

Time of exposure is another critical aspect of antimicrobial effectiveness and is even more important when it comes to biofilm eradication. Most studies center that have been conducted to assess irrigants has focused on its ability to eradicate free-floating planktonic bacteria, termed minimum inhibitory concentration (MIC). A study done in 2022 explored the minimum effective exposure time required to prevent growth of Staph aureus, Staph epidermidis, and Cutibacterium acnes with antiseptic solutions commonly used in arthroplasty and found successful eradication within 120 s (50). However, this study also used planktonic bacterial cultures, which does not take into consideration how this epxosure time may be affected by biofilm. The minimum biofilm eradication concentration (MBEC) *in vitro* can be hundreds to thousands times higher than the MIC for the exact same microorganism in its planktonic form (51, 52). This can manifest as a crucial difference when it comes to clinical significance, as bacterial persister cells within the biofilm may survive and repopulate the biofilm, which can explain the prevalence of recurrent recalcitrant biofilm formation and subsequent PJI. Castaneda et al. investigated the importance of antimicrobial exposure time and found a positive correlation between biofilm susceptibility to antimicrobials up to 32-fold when continuously exposed to antimicrobials for longer time (53).

The effectiveness of citrate-based solutions is further enhanced by their prolonged exposure time. This important difference is highlighted in the *in-vitro* study conducted by Bashyal, where because the citrate-based solution (XP) did not require a secondary washout, it was allowed up to 5 h of contact time *in vivo*, reducing biofilm production by up to 8-log in that time period. On the other hand, due to host cytotoxicity, PI, Irrisept, and Vashe were all rinsed out after no more than 5 min of exposure (42).

## 9 Decreased cytotoxicity

**Figure 1:**
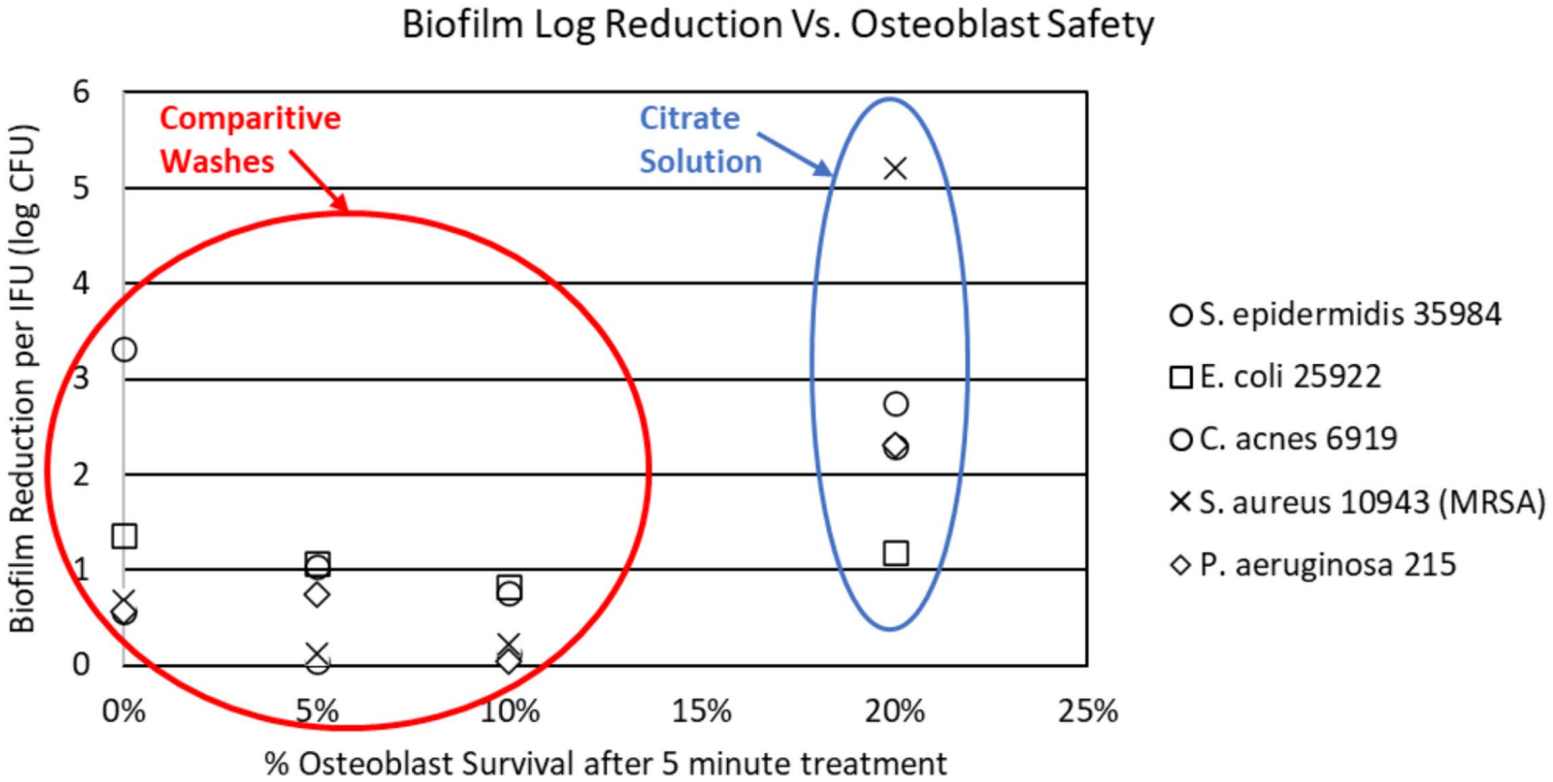
Citrate solution compared to routinely used irrigation solutions in biofilm reduction and osteoblast survival.

This extended exposure time is also attributed to XP’s low toxicity on native cells and host tissue. Dr. Boyle Cheng’s osteoblasts safety presentation at Disasterplasty (54) compared the osteoblast safety of several wash solutions for surgical irrigation in arthroplasty. In this testing, he demonstrated that of the antimicrobial washes, the citrate solution induced the least osteoblast destruction at 5 min and 24 h of exposure. Additional testing confirmed that osteoblasts exposed to the citrate solutions were able to mineralize at a much higher concentration of application than the comparative products. Figure 1 demonstrates the superior efficacy of a citrate-based solution in comparison to other routinely used washes (PI, Irrisept, Vashe) in regards to biofilm reduction and osteoblastic safety profile.

To determine if the low toxicity of the citrate solution had additional patient advantages, Dr. Andrew Wickline performed a small pilot study comparing post-operative inflammation after TKA in patients irrigated with this citrate-based solution versus standard sterile dilute iodine lavage. In this testing, he demonstrated that patients washed with the citrate solution as a final wash had lower inflammation in the 7 to 21 day post-op period, which ultimately led to lower pain scores and opiate use, increased range of motion, and an earlier return to unassisted walking (55).

Furthermore, citrate has shown high rates of release of bone morphogenic protein (BMP), TGF-β1, VEGF, and IGF (37), all essential growth factors in the regeneration and reparation of bone via several mechanisms, including modulation and balance of osteoblastic and osteoclastic function (56), hence its routine usage in regenerative endodontic procedures (37). This induction of release of growth factors may potentially explain why citrate has less cytotoxic activity on human osteoblastic cells (57). Additionally, targeting the dissolution of biofilm structure versus eukaryotic cells may further explain why this solution is purported to have lower toxicity while having higher efficacy.

## 10 Point of attack: clinical use of a citrate-based solution

The use of the XP as a citrate-based irrigant can be utilized in a variety of ways for the practicing clinician. Its current use is primarily maximized in the operating theater. Applications in orthopedic and podiatric surgery are presently where it sees its highest volume of use. Critical to the success of the variety of surgeries performed is the need for effectual, clinically proven, scientifically grounded treatments that are above all safe for patients.

As noted in this paper, avoidance of harmful soft tissue injury at a cellular level is critical when using these varieties of solutions available in the orthopedic marketplace. The citrate-based irrigant XP seems to fit this narrow window of management in this treatment space.

Orthopedic-related trauma with open fracture management is clearly an important and effective reason for its application. The composition of this citrate-based irrigant is quite effective against the variety of organisms that are encountered in the acute management of bone and soft tissue trauma (42).

Elective or cold trauma surgery is another opportunity for its use as a preventative solution for potential peri-operative infection. As previously described, the incidence of PJI is a growing concern and the costs associated with its management on the whole are mind-boggling.

In the following discussion, details of the typical steps associated with the use of the XP citrate-based solution in a routine primary joint replacement will be highlighted. The solution in our respective institution is placed within a basin on the sterile back table. Typically, *Asepto* bulb syringes are used for irrigation. In the case of hip arthroplasty surgery, whether a primary or revision surgery, wound towels along the margins of the wound are soaked with the solution and placed under retractors for the remainder of the surgery. Further dissection into the joint is performed and the liberal use of the irrigant is encouraged during the remainder of the individual surgeries. The critical use of this solution may perform at its best when associated with performing bone cuts. Upon completion of these cuts either hip or knee, the cancellous bone should be lavaged. The amount should be the individual surgeon’s choice. Following this, in the knee, patting the bone surfaces versus suctioning should be considered. Simple suctioning for hip surgery should suffice while maintaining some solution within the hip cavity.

For cementless implant fixation (hip or knee), lavage of the porous surfaces should be considered. Please see our prior discussion on the safety associated with the use of this on cancellous bone and the maintenance of the osteocyte/osteoblast. The potential for enhanced fixation of the implant will have tremendous value as well. With the bone surfaces and implants enhanced with the irrigant, the final implantation can proceed.

For purposes of closure, both deep and superficial layers are to be rinsed, in our hands two full *Asepto* syringes are emptied into the joint space, and upon closure, repeat lavage of the superficial layer ensues. Alternatively, the solution can be instilled with mechanical high-pressure mechanical lavage systems.

Skin closure is followed with the application of *Surgx_TM_*, an antimicrobial gel designed to reduce surgical site and post-surgical infections based on the same technology as XP.

## 11 Future studies exploring citrate-based solutions

Most of the literature on the antimicrobial properties and usage of citrate in the treatment of infections pertains to studies done either *in vitro* or outside of the orthopedic setting, such as in the aforementioned fields of endodontics and ophthalmology, for example. Therefore, it cannot be safely assumed that a citrate-based solution would either more effectively mitigate PJI risk or treat an existing PJI when compared to other standard lavages.

*In-vitro*, while many studies have focused on MIC, more studies could focus on establishing the MBEC of citrate-based solutions, which may present a more accurate assessment of its efficacy in the treatment of PJI. On a more molecular level, sub-inhibitory concentrations of citrate-based solution and its effect on *ica* gene expression (and consequently PIAs) in *S. aureus,* the most commonly implicated pathogen in PJI, could further support its use, since it is well established that eradication of EPS, and in particular PIA, is a crucial step in biofilm penetrance.

Perhaps more importantly, more large-scale *in-vivo* analyses of the usage of citrate-based solutions in the treatment of PJI need to be conducted. Greater statistical strength can be obtained from retrospective analyses such as the one done at Jack Hughston Memorial Hospital (58) and Edgewater Surgical Center (59). However, both of these studies lacked comparison to a comparable control cohort that did not receive XP. While another retrospective analysis expanding on these existing studies with the inclusion of a control group may be a simple way to more accurately assess potential superior performance of the citrate-based solution XP, a well-controlled prospective analysis comparing the efficacy of a citrate-based solution versus the current standard therapies would yield the most reliable results.

## Data availability statement

The original contributions presented in the study are included in the article/supplementary material, further inquiries can be directed to the corresponding author.

## Author contributions

DV: Writing – original draft, Writing – review & editing. JM: Writing – original draft, Writing – review & editing.
